# Time-domain transmission sensor system for on-site dielectric permittivity measurements in soil: A compact, low-cost and stand-alone solution

**DOI:** 10.1016/j.ohx.2023.e00398

**Published:** 2023-01-31

**Authors:** Manuel Pérez, Diego Mendez, Diego Avellaneda, Arturo Fajardo, Carlos I. Páez-Rueda

**Affiliations:** Pontificia Universidad Javeriana, Bogotá D.C. 110231, Colombia

**Keywords:** Dielectric permittivity, Dielectric measurements, Time-domain transmission, Soil water content

## Abstract

Dielectric-based measurement techniques have been shown to be very effective in determining the properties of various materials. These techniques have been widely used in a variety of fields and applications. Time Domain Transmission (TDT) techniques have grown in popularity because they are practical, non-destructive, provide measurements in real time and produce accurate measurements that are independent of multiple reflections. TDT techniques, on the other hand, are mostly performed with specialized bulky laboratory equipment, such as a Vector Network Analyzer (VNA) which makes TDT measurements prohibitively costly and unpractical. In fact, few works in the literature have reported portable on-site TDT systems. The aim of this paper is to design and implement a dedicated, compact, and low-cost microwave Time Domain Transmission (TDT) sensor for measuring superficial soil dielectric properties on-site. Our sensor uses a time-delay measurement technique over a microstrip transmission line to estimate the dielectric properties of the soil under test. Measurement results show that the computed mean absolute error (MAE) is less than 1.2 when compared to a calibrated dielectric assessment kit (DAK) with soils containing less than 20 % of water ($\varepsilon \prime_{r}<5.0$), implying that our TDT sensor system can obtain on-site measurements in relatively dry soils with acceptable accuracy.

## Graphical abstract

**Figure:**
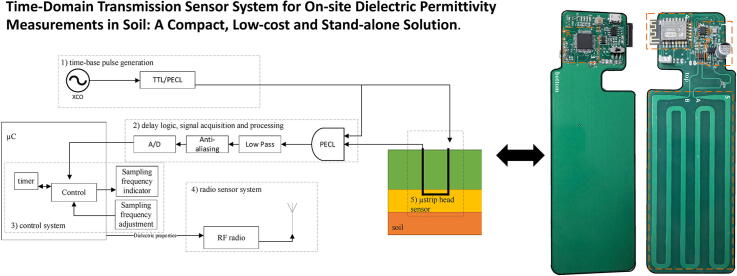

**Specifications table**

**Table:** 

**Hardware name**	*TDT Sensor*
**Subject area**	• Engineering and material science
**Hardware type**	• Measuring physical properties and in-lab sensors • Field measurements and sensors
**Closest commercial analog**	No commercial analog is available.
**Open source license**	CC BY 4.0
**Cost of hardware**	$ 60 USD
**Source file repository**	https://data.mendeley.com/datasets/mt24hdxsr2/1
**Project DOI URL**	https://doi.org/10.17632/mt24hdxsr2.1

## 1 Hardware in context

There is a particular need to accurately estimate the properties of soil materials in a wide variety of applications. Humanitarian demining, precision agriculture, and geological and hydrological applications, for example, necessitate rapid and on-site soil properties measurements. In humanitarian demining and geological applications, the used equipment and tools require accurate calibration, which is usually determined by the specific properties of the soil under test. Similarly to hydrological applications and precision agriculture, the precision of the AI-based algorithms used is heavily dependent on the precise properties of the soil being used.

Dielectric-based measurement techniques have been shown to be very effective in determining the properties of various materials. These techniques have been widely used in a variety of fields, including the development of novel sensor systems. Dielectric measurement methods rely on determining the relationship between the environment’s complex dielectric permittivity and the material properties. When changing the physical properties of a material, these indirect measurements have proven to be very precise and reliable. Furthermore, current dielectric-assessment techniques are frequently prohibitively expensive, impractical, time-consuming, destructive, labor-intensive, and require bulky equipment. Many of the aforementioned applications require on-site rapid measurement of material dielectric properties.

Time Domain Reflectometry (TDR) methods are frequently used to determine the dielectric properties of various materials [1], [2]. TDR methods, like other microwave techniques, have grown in popularity because they are practical, non-destructive, and provide measurements in real time. Furthermore, TDR methods are based on the transmission and time-analysis of baseband signals, which improves sensitivity against material water content. Reflectometry methods, on the other hand, are limited due to multiple reflections caused by inhomogeneities in the Material Under Test (MUT). Time Domain Transmission (TDT) techniques, on the other hand, produce accurate measurements that are independent of multiple reflections because the transmitted impulse signal is slightly influenced by scattered signals and is easily identified in the signal processing stage. When compared to TDT systems, the work in [3] shows that the accuracy of a TDR system decreased due to multiple reflections caused by inhomogenities in the surrounding material. As a result, TDT systems provide advantages when measuring dielectric properties in inhomogeneous materials such as soil. TDT techniques, on the other hand, are mostly performed with specialized laboratory equipment, such as a Vector Network Analyzer (VNA) [4]. Although some solutions based on other equipment, such as the Pocket VNA 1, could offer an acceptable reading, it is not a compact and standalone solution as the one proposed here.

Only a few works [5], [6] have reported portable on-site TDT systems. The work in [7] proposes a TDT microwave soil moisture sensor operating at 1.4 GHz with a sensor probe made of a microstrip transmission line. However, the sensor has operational drawbacks because it uses a phase detector to measure the phase difference between the reference and the delayed signal received from the microstrip line, which causes phase ambiguity and reduces the sensor’s dynamic range. Furthermore, the generated signal is limited to a single tone at 1.4 GHz, which limits the estimation of dielectric properties in broadbands.

The authors of [5] introduced a compact cost-effective TDT measurement system with very accurate and stable measurements; however, some concerns have been raised regarding the complexity of the implementation of the sensor head via a concentric reversion coupler, which makes the sensor deployment impractical and costly. Furthermore, the sensor measurement setup requires the introduction of a plastic tube into the soil, which complicates the measurement for two reasons: first, the measurement setup can be impractical and difficult, and second, there is an air gap within the tube, which can eventually modify the measured dielectric properties of the soil.

The aim of this paper is to design and implement a dedicated, compact, and low-cost microwave Time Domain Transmission (TDT) sensor for measuring superficial soil dielectric properties on-site. Our sensor uses a time-delay measurement technique over a microstrip transmission line to estimate the dielectric properties of the soil under test in an indirect manner. The final sensing device combines the sensor head, a processor unit, a power management module, and a wireless transmitter to enable on-site and stand-alone soil sample characterization, resulting in a comprehensive and scalable solution.

## 2 Hardware description

To achieve a stand-alone solution, the proposed system fully integrates all required components, including the microstrip head sensor. As a result, no external radio-frequency connectors or other interference-sensitive components are required for the measurement to be performed. As a result, a compact low-cost TDT sensor for permittivity measurements is presented, which has been optimized for various rapid on-site, non-destructive surface soil measurements and can be adapted for a variety of applications.

### 2.1 General architecture

**Fig. 1:**
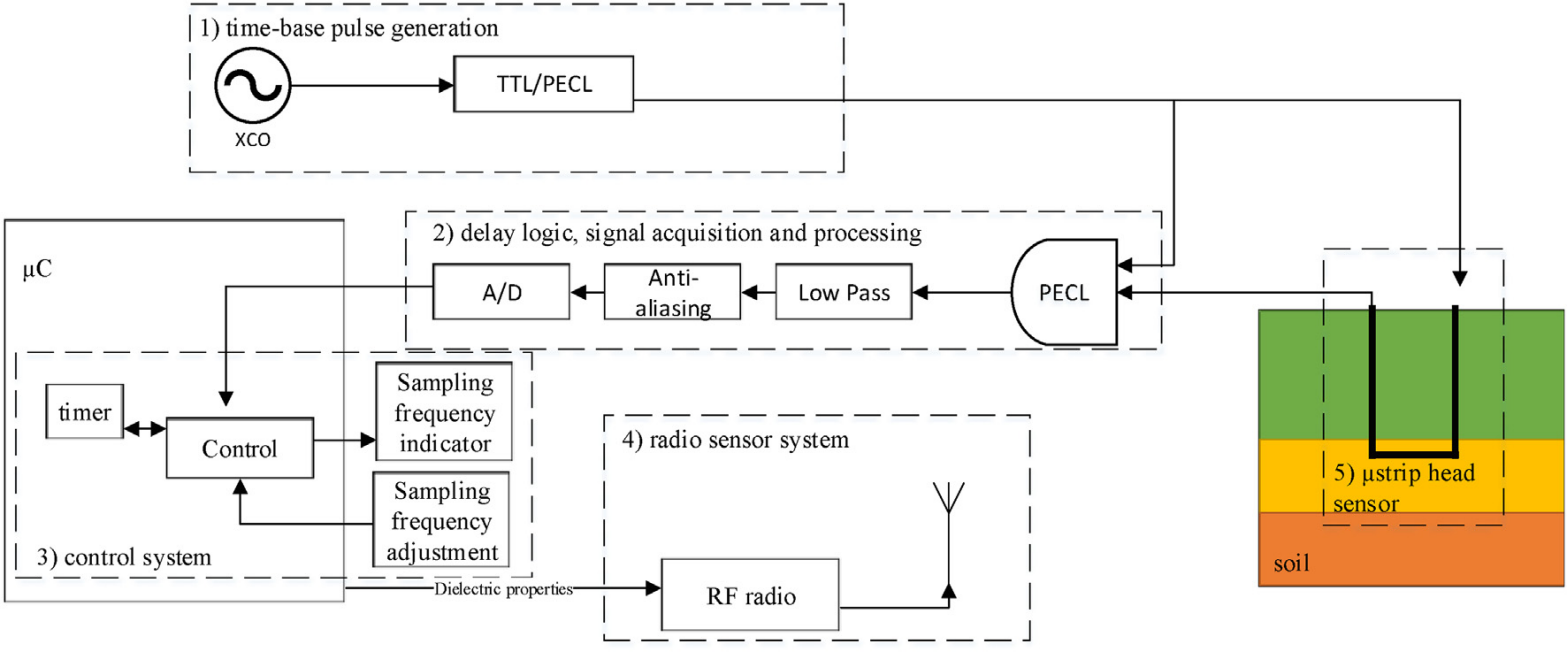
System architecture of the TDT system subdivided into 1) a time-base pulse generator, 2) the delay logic including the signal acquisition and processing units, 3) the control system unit, 4) the radio sensor system and 5) the microstrip head sensor.

Fig. 1 presents the system architecture of the TDT sensor system, which is divided into five major components: (1) time-base pulse generation, (2) delay logic including signal acquisition and processing, (3) control system, (4) radio sensor system, and (5) microstrip head sensor. A square wave oscillator with an oscillation frequency of 50 MHz is used to generate time base pulses. Since the maximum designed delay was 10 ns when considering a 50% duty cycle at the oscillator, no synthesizer was considered for the oscillator to decimate the frequency. The chosen oscillator has low jitter, resulting in good frequency and pulse-width stability and higher precision.

A Positive Emitter-Coupled Logic (PECL) high-speed AND gate is used as a novel component of our system to compare the generated input signal, which serves as a reference, and the generated output signal coming from the sensor head (receiving port B), which will be called the TDT signal. As a result, time-domain measurements are performed as the primary acquisition concept. Because the transistors never operate in saturation mode, the input/output voltage swing is small (0.8 V), the input impedance is high, and the output impedance is low, the PECL technology was chosen. As a result, transistors change states quickly (in the order of picoseconds [ps]), gate propagation delays are short, and fanout capability is high. Furthermore, because the chosen oscillator employs transistor-transistor logic (TTL) technology, a TTL to differential PECL translator was considered before passing the reference signal through the transmitting port (A) of the microstrip head sensor and the PECL AND gate.

**Fig. 2:**
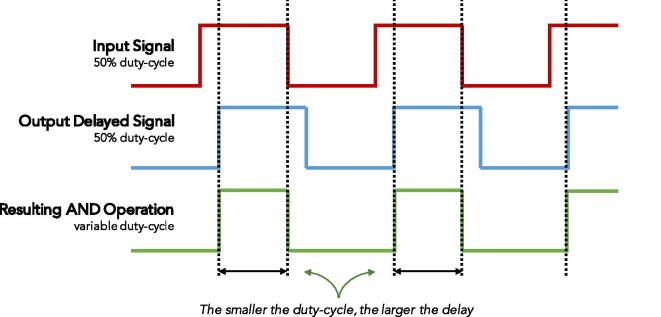
A representation of the input squared signal that feeds the sensor (top in red), the delayed wave that generates the sensor (middle in blue), and the resulting signal after the AND operation is performed (bottom in green).

The pulse with modulated (PWM) signal is produced by the PECL AND gate, which compares the reference signal and the TDT signal. The duty cycle of the PWM signal contains all the delay information. Fig. 2 shows a representation of the input signal that feeds the sensor (red), the delayed output signal generated by the sensor (blue) and the resulting signal after performing the AND operation (green). As can be seen, the delay, which will later be associated with the complex permittivity of the sample, increases as the resulting duty-cycle decreases.

To obtain an average DC value, the resulting PWM signal is low-pass filtered. To extract the delay information, the cutoff frequency, order, and frequency response of the low-pass filter were adjusted. Finally, before sending the low-passed signal to the analog-to-digital converter (A/D), it is passed through an anti-aliasing filter to limit the signal bandwidth and avoid interference with the A/D converter’s sampling process. This filter must be designed based on the sampling frequency and the low-passed signal input. The signal is processed in the microcontroller (control system) after the A/D conversion stage, where the control and communication radio systems operate.

The A/D signal is acquired by the control system based on a sampling frequency defined by the user via an interface. When the control system reads the A/D signal, the measurement is sent to a computer station via the radio system using Universal Asynchronous Receiver-Transmitter (UART) serial communication. According to the communication requirements of the specific application, the radio system was designed to operate via the WiFi network. In order to perform soil permittivity measurements, the general information of the sensor node and the delay obtained from the microstrip head sensor are combined.

### 2.2 TDT sensor design

**Fig. 3:**
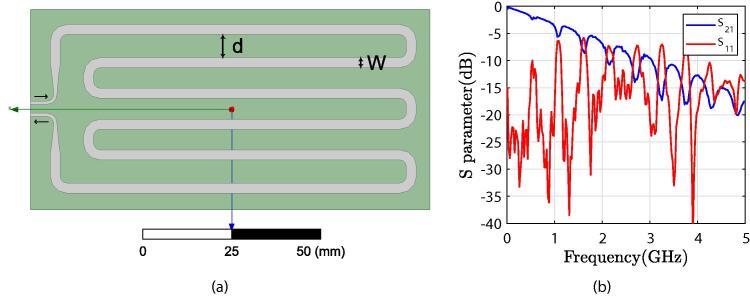
(a) TDT microstrip line sensor head model and (b) Measured reflection and transmission parameters of the microstrip head sensor.

In the case of a very humid soil sample, a compact microstrip sensor design allows TDT measurements for water–solid materials mixtures. In comparison to reflection-based measurements, TDT sensors have the advantage of not being affected by multiple reflections, as previously stated. However, one major concern about sensor design is considering both sensor ends on one side. To meet this requirement and maintain the sensor’s compactness, a meander technique is used, in which the coupling between adjacent microstrips is primarily determined by the distance *d* between the lines, as shown in Fig. 3a. The distance *d* and width *W* of the microstrip were numerically optimized to increase the time-delay resolution using Ansys Electronics software to improve performance in terms of return and transmission losses for an operating frequency ranging from DC to 2 GHz, thus covering the frequency range containing the spectral content of the TDT signal. The optimized values (d=6.5mm,W=2.8mm and L=73.6mm) over a glass-reinforced epoxy laminate material (FR4) substrate result in a line impedance of $Z_{0}=50\;\Omega$, resulting in a reflection coefficient parameter $S_{11}⩽-10$ dB and a transmission $S_{21}⩾-10$ dB for baseband signals up to 2 GHz. Measured reflection and transmission scattering parameter of the miscrostrip head sensor are presented in Fig. 3b. When the delay time of the microtrip line is long, as shown in [8], the sensor’s resolution improves. The chosen sensor delay time on air was 6 ns as a trade-off between sensor resolution and sensor compactness.

### 2.3 Mathematical model

A Pulse-width modulation (PWM) technique is used at the delay logic, signal acquisition and processing block in Fig. 1. Thus, the output signal of the PECL AND gate is pulse modulated and contains all the delay information through its duty cycle. The PWM output signal $f_{\mathrm{PWM}}(t)$ from the PECL AND gate, with period *T*, low value $V_{\min}$, high value $V_{\max}$ and a duty cycle *D*, is low-passed filtered to obtain the average DC value which is given by (1).

(1) $${\overset{‾}{V}}_{\mathrm{PWM}}=\frac{1}{T}\int_{0}^{T}\;f_{\mathrm{PWM}}(t)\;\mathrm{dt}$$

As $f_{\mathrm{PWM}}(t)$ is a pulse signal, its values is $V_{\max}$ for 0<t<D·T and $V_{\min}$ for D·T<t<T. Equation in (1) can be easily expressed as:

(2) $${\overset{‾}{V}}_{\mathrm{PWM}}=D\cdot V_{\max}+(1-D)\cdot V_{\min}$$

From (2), the duty cycle value can be obtained from the ${\overset{‾}{V}}_{\mathrm{PWM}}$ value and the maximum and minimum voltage range of the logic levels in the low-voltage PECL technology which are $V_{\min}=[1.54\;V,1.70\;V]$ and $V_{\max}=[2.09\;V,2.25\;V]$. The ${\overset{‾}{V}}_{\mathrm{PWM}}$ voltage is obtained from the A/D converter with a resolution given by (3).

(3) $$Q=\frac{E_{\mathrm{FSR}}}{2^{M}}$$

where M is ADC’s resolution in bits and $E_{\mathrm{FSR}}$ is the full scale voltage range.

As can be easily deduced from Fig. 2, the measured delay time can be obtained from the off time of the AND output signal and is given by (4).

(4) $$t_{\mathrm{delay}}=(1-D)\cdot T$$

where *T* is the period of the AND signal, this is given by considering half of the period of the 50 MHz pulse train generated signal.

The proposed TDT sensor system makes use of the relation between the delay time of the electromagnetic waves and the complex dielectric permittivity ε^=ε0ε^r=ε0(εr′-i·εr′′) of the surrounding material, where $\varepsilon_{0}$ is the free space permittivity. On one hand, the real part $\varepsilon_{r}^{\prime }$ takes in consideration the energy stored in the dielectric material at a given frequency and temperature. On the other hand, the imaginary part $\varepsilon_{r}^{\prime \prime }$ describes the dielectric losses or the energy dissipation. Therefore, the relative complex dielectric permittivity at a given frequency *f*, can be written as:

(5) ε^r=εr′-i·εrelax′′+σdc2πfε0

In (5), the imaginary part of the expression expresses the losses, which are associated with two main processes: molecular relaxation ($\varepsilon_{\mathrm{relax}}^{\prime \prime }$) due to the relaxation time required by a dipole to adjust its orientation according to the components of the applied electromagnetic field, resulting in absorption of energy by the dipole, and the electrical conductivity ($\sigma_{\mathrm{dc}}$) due to conduction arising form the material surfaces as a result of electrical charges.

Time domain transmission techniques measures the propagation velocity of a transmission pulse. The velocity of the signal is primarily a function of the permittivity of the material through which it travels with potential modification by conductive losses. From the material dielectric properties, electric permittivity ε and magnetic permeability μ, the wave velocity of a propagating electromagnetic field is given by:

(6) $$v_{p}=\frac{1}{\sqrt{\mu \cdot \varepsilon }}=\frac{1}{\sqrt{\left(\mu_{0}\;\varepsilon_{0})\cdot (\mu_{r}\;\varepsilon_{r}\right)}}=\frac{c_{0}}{\sqrt{\mu_{r}\;\varepsilon_{r}}}$$

where $c_{0}$ is the velocity of light and $\mu_{0}$ is the free space permeability.

From the mechanical point of view, the velocity of an electromagnetic wave travelling through a line probe of length *L* is given by (7).

(7) $$v=\frac{L}{t_{\mathrm{delay}}}$$

where $t_{\mathrm{delay}}$ is the measured time delay in the TDT system from (4). By defining the relation between the propagating wave velocity in (6) and the free space velocity $c_{0}$, it can be easily found the following relation:

(8) $$\frac{1}{\sqrt{\mu_{r}\varepsilon_{r}}}=\frac{L}{c_{0}\;t}$$

where $\mu_{r}$ is the relative permeability, since most of the soils can be considered a non-magnetic, $\mu_{r}=1$ [], therefore Eq. (6) can be rewritten as:

(9) $$\varepsilon_{r}={\left(\frac{c_{0}\;t_{\mathrm{delay}}}{L}\right)}^{2}$$

The Eq. (9) constitutes a basis for TDT analysis to obtain the relative dielectric permittivity, where the time delay is measured, and the probe length and the wave velocity are known.

The primary assumption in for dielectric time-based measurements is that imaginary part of the complex relative dielectric permittivity is negligible thus ε^r≈εr′. However, although small, dielectric losses are always present and therefore time measured dielectric permittivity was called apparent permittivity $\varepsilon_{a}$. The contribution of non-accounted imaginary component to the measured apparent dielectric permittivity, results into an overestimation of the real component of the electric permittivity $\varepsilon_{r}^{\prime }$. The apparent permittivity $\varepsilon_{a}$ is given by:

(10) $$\varepsilon_{a}=\frac{\varepsilon_{r}^{\prime }}{2}\left(1+\sqrt{{\left(\frac{\varepsilon_{r}^{\prime \prime }}{\varepsilon_{r}^{\prime }}\right)}^{2}+1}\right)$$

### 2.4 Sensor Calibration

For calibration purposes, the physical probe length *L* in (7) is taken as the length of the obstacle $L_{\mathrm{obs}}$, which due to the influence of the surrounding material in the sensor boundary areas, it has variations so a so-called effective obstacle length $L_{\mathrm{obs},\mathrm{eff}}$ is computed to archive precise measurements. In this regard, a two-point calibration procedure with two different known permittivities ($\varepsilon_{\mathrm{cal}}$ and $\varepsilon_{\mathrm{ref}}$) is performed. Therefore, a calibration factor α is computed in (11) for obtaining the relation between $L_{\mathrm{obs}}$ and $L_{\mathrm{obs},\mathrm{eff}}$ in (12).

(11) $$\alpha =(\sqrt{\varepsilon_{\mathrm{cal}}}-\sqrt{\varepsilon_{\mathrm{ref}}})\cdot \frac{1}{t_{\mathrm{cal}}-t_{\mathrm{ref}}}\cdot \frac{L_{\mathrm{obs}}}{c_{0}}$$

(12) $$L_{\mathrm{obs},\mathrm{eff}}=\frac{L_{\mathrm{obs}}}{\alpha }$$

### 2.5 Measurement on unknown material

Measurement of unknown material are based on the previous measurement of a reference material with known permittivity $\varepsilon_{\mathrm{ref}}$ which yields a reference delay time $t_{\mathrm{ref}}$. In consequence, the delay time difference of the reference material and the material under test (MUT) can be used to determine its apparent permittivity $\varepsilon_{\mathrm{MUT}}$ by using the effective obstacle length $L_{\mathrm{obs},\mathrm{eff}}$ through:

(13) $$\varepsilon_{\mathrm{MUT}}={\left(\frac{(t_{\mathrm{meas}}-t_{\mathrm{ref}})\cdot c_{0}}{L_{\mathrm{obs},\mathrm{eff}}}+\sqrt{\varepsilon_{\mathrm{ref}}}\right)}^{2}$$

By computing the apparent permittivity of the MUT through (13), only the permittivity of the surrounding material of the sensor and the effective length of the obstacle are taking into account. Neither the measuring position nor permittivities in other regions along the measured profile of the head sensor have any influence on the the measurement.

### 2.6 Schematic diagram and relevant features

**Fig. 4:**
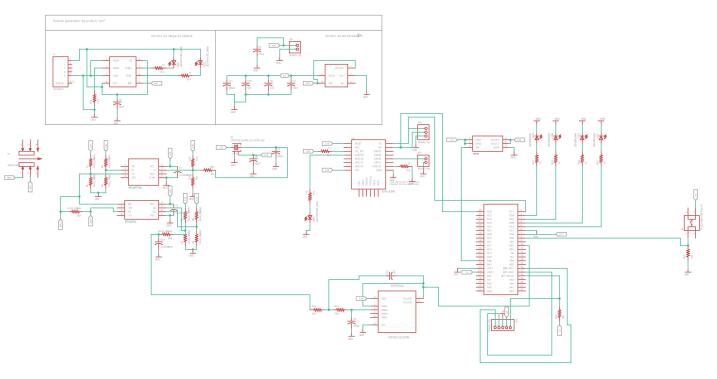
Schematic diagram of the TDT sensor system.

The schematic diagram for the TDT sensor system in shown in Fig. 4. Some of the main components in the design were: The Micro-controller PIC18F45K22-I/PT from Microchip Technology, that was used to implement the control system unit. For the delay logic unit, the 2-input differential AND/NAND gate MC10/100EP05 from Semiconductor. The WiFi ESP8266-SMD module was used for the radio system unit.

The Printed Circuit Board (PCB) was designed in the software Altium Designer, resulting in a board size of 168 mm × 55 mm. The most relevant features of the TDT sensor system are listed below:

• It allows stand-alone operation for rapid on-site, non-destructive dielectric measurements of different soils.
• It is low cost, portable and easy to install.
• Because its WiFi connectivity, the system can be integrated into a wireless sensor system for a massive sensor deployment in field within a communication range of 500 m in line of sight.

## 3 Design files

Table 1 lists the design files associated to the proposed TDT sensor system. The repository 2 contains the files needed to implement the TDT sensor system. These files are organized in folders as follows:

• *Board*_*Fabrication*_*Files* folder: Contains the Gerber files, bill of Materials (BoM), the schematic (.sch) and the board (.brd) files to produce the electronic board of the TDT sensor.
• *ESP8266*_*TDTSensor*_*Firmware* folder: Contains the source code developed in Arduino IDE used to program the ESP8266 WiFi module.
• *PIC*_*TDTSensor*_*Firmware* folder: Contains the source code project developed C++ used to program the MCU PIC18F45K22 module through MPLAB IDE.
• *Post-Processing* folder: Contains the source code files developed in Matlab, for computing the sensor calibration and computing of the time delay and dielectric permittivity.
• *General*_*Design*_*Figures* folder: Contains the Figures included in this paper.

**Table 1:** Design files of the proposed TDT sensor.

**Design filename**	**File type**	**License**	**Location of the file**
General Architecture	PDF Figure	CC BY 4.0	Included in the paper (Fig. 1)
Schematic diagram	PDF Figure	CC BY 4.0	Included in the paper (Fig. 4)
Assembled TDT sensor system	PDF Figure	CC BY 4.0	Included in the paper (Fig. 5)
Connection Diagram for ESP8266 Programming	PDF Figure	CC BY 4.0	Included in the paper (Fig. 6a and 6b)
Connection Diagram for MCU Programming	PDF Figure	CC BY 4.0	Included in the paper (Fig. 7)
TDT sensor system operation	PDF Figure	CC BY 4.0	Included in the paper (Fig. 8)
Measurement set-up for TDT sensor system validation.	PDF Figure	CC BY 4.0	Included in the paper (Fig. 9a and 9b)

## 4 Bill of materials

The list of materials used in the design of the TDT sensor system is presented in Table 2. All the electronic components can be acquired in different electronics stores such as Digikey, Mouser or Arrow. The purchase links can be found in the BoM spreadsheet located in the Board_Fabrication_Files folder within the repository presented in the previous section.

**Table 2:** Bill of Materials of the TDT sensor system.

**Designator**	**Component**	**#**	**Cost unit USD**	**Total cost USD**	**Source**	**Material type**	
Resistor	1.2 kΩ±0.1% 0.1 W, C0603 SMD	5	$ 0.35	$ 1.75	Digikey	Other	
Resistor	1 kΩ±5% 0.25 W C0603 SMD	10	$ 0.1	$ 1	Digikey	Other	
Resistor	220 Ω±0.1% 0.1 W C0603 SMD	5	$ 0.1	$ 0.5	Digikey	Other	
Resistor	330 Ω±5% 0.1 W C0603 SMD	5	$ 0.1	$ 0.5	Digikey	Other	
Resistor	50 kΩ±0.5% 0.63 W C0603 SMD	5	$ 0.14	$ 0.7	Digikey	Other	
Capacitor	10 *μ*F ±20% 6.3 V C0603 SMD	20	$ 0.2	$ 4	Digikey	Ceramic	
Capacitor	100 pF ±5% 50 V C0603 SMD	5	$ 0.1	$ 0.5	Digikey	Ceramic	
Capacitor	1 *μ*F ±20% 16 V C0603 SMD	20	$ 0.1	$ 2	Digikey	Ceramic	
Micro-controller	IC MCU 8bit 32 kb Flash 44TQFP	1	$ 3.76	$ 3.76	Digikey	Semiconductor	
Logic Gate	IC Gate AND/NAND ECL 2INP 8SOIC	1	$ 11.91	$ 11.91	Digikey	Semiconductor	
TTL/CMOS-PECL translator	IC TRNSLTR UNIDIRECTIONAL 8SOIC	1	$ 10.48	$ 10.48	Digikey	Semiconductor	
Radio Module ESP8266	WiFi 802.11b/g/n TxRx Mod. 2.4 GHz-2.5 GHz TSM	1	$ 6.95	$ 6.95	Digikey	Semiconductor	
Crystal	50 MHz HCMOS XO 3.3 V	1	$ 1.12	$ 1.12	Digikey	Other	
Power Switch/Driver	PWR Switch N-CHAN 1:1 6LFCSP	1	$ 1.72	$ 1.72	Digikey	Semiconductor	
OP Amplifier	IC OpAmp GP 2 Circuit 8SOIC	1	$ 1.31	$ 1.31	Digikey	Semiconductor	
Regulator	IC linear regulator 3.3 V 1A SOT25	1	$ 0.96	$ 0.96	Digikey	Semiconductor	
LED	Blue 470 nm LED 2.8 V C0603	5	$ 0.3	$ 1.5	Digikey	Other	
Tactile Switch	SPST-NO 0.02A 15 V SMD	1	$ 0.65	$ 0.65	Digikey	Other	
Switch	Slide Switch 300 mA 6 V SMD	1	$ 0.58	$ 0.58	Digikey	Other	
USB Connector	Micro B USB 2.0 Receptacle Connector 5 SMD, Right Angle	1	$ 0.48	$ 0.48	Digikey	Other	
Battery Connector	CONN HEADER VERT 2Pos 2.5 mm	1	$ 0.14	$ 0.14	Digikey	Other	
Li-ion Battery	Bat. 3.7 V 400mAH	1	$ 5.5	$ 5.5	Digikey	Other	

## 5 Build instructions

**Fig. 5:**
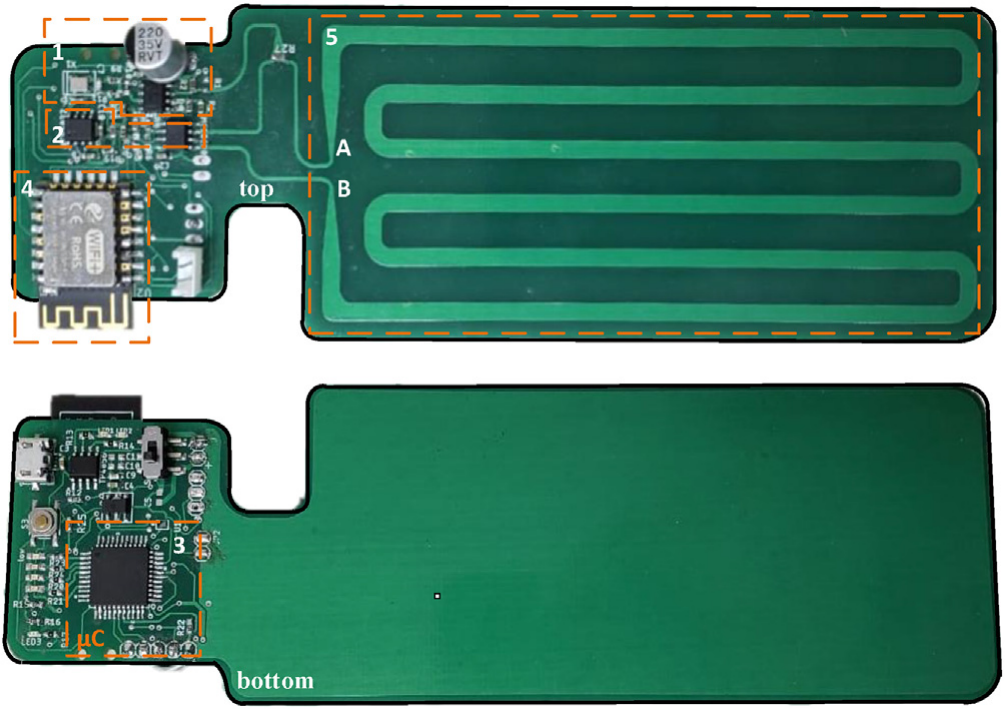
Assembled TDT sensor system on a two-layer PCB by using an stencil and a reflow oven after manually placing the electronics components. In the Figure it is shown the measuring ports (A and B) and and the following functional components: (1) the crystal oscillator XCO for the pulse train generation, (2) a low voltage PECL AND gate and low pass filter, (3) the microcontroller, (4) the WiFi radio module and (5) the miscrostrip head sensor.

For implementing the TDT sensor system, two option are possible, either to use a specialized electronics manufacturing service which require the Gerber and the BoM files which are included in the Board_Fabrication_Files folder of the repository. The fabrication cost for this option is around $ 45 USD for double side placement of 1 piece without including the cost of the electronics components and shipping. The second option is to use only a specialized PCB fabricator. Prices for fabrication of 5 pieces with the included option of stencil is around $ 15 USD without the shipping cost. Soldering of electronics components on the PCB can be perform with the use of soldering paste with the stencil and after manually placing the components, the use of a reflow oven. In the case of missing a reflow oven, soldering the manually placed electronics components with a station is also possible by employing soldering flux and paste. The station should be set to 250 °C and a level tip of 2.1 mm width. An assembled prototype of the TDT sensor system is shown in Fig. 5.

With the TDT sensor system board assembled, next step is to load the firmware file into the ESP8266 control unit (ESP8266MCU_TDTSensor_Firmware.ino) included in the ESP8266_TDTSensor_Firmware folder in the repository. Before loading the ESP8266 firmware it is important to set the Single Service Identificator (SSID) and the password of the WiFi network. The loaded firmware can be adapted to send the information to different platforms through the MQTT protocol, using the ”PubSubClient.h” library. All reported data is visualized through Ubidots platform, however other MQTT broker can be also used. This task requires the Arduino IDE application.

**Fig. 6:**
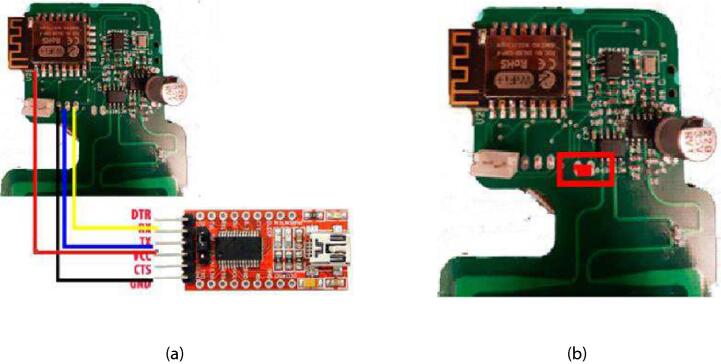
Connection diagram for the ESP8266 firmware loading: (a) FTDI TTL-Serial connection and (b) Connection of the DuPont jumper.

For loading the ESP8266 firmware an FTDI TTL-Serial converter with logic levels set to 3.3 V must be used and connected as shown in Fig. 6a. In addition, during the loading the DuPont jumper should be connected as indicated in Fig. 6b. Once connected, in the Arduino IDE you must choose the port that corresponds to the FTDI and the Generic ESP8266 as the card. After compiling and loading the program, the DuPont jumper must be removed for running the program.

**Fig. 7:**
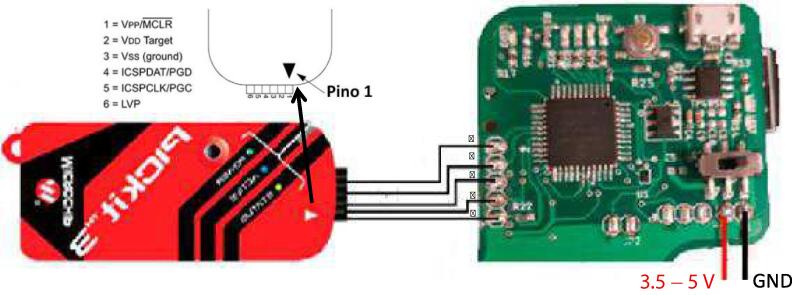
Programming of the MCU of the TDT sensor system.

For loading the PIC 18F45K22 firmware the project PIC.X included in the PIC_TDTSensor_Firmware folder of the repository must be uploaded via the PICkit3 programmer. Connection for uploading the PIC firmware are depicted in Fig. 7. This task requires the MPLAB IDE application.

## 6 Operation instructions

**Fig. 8:**
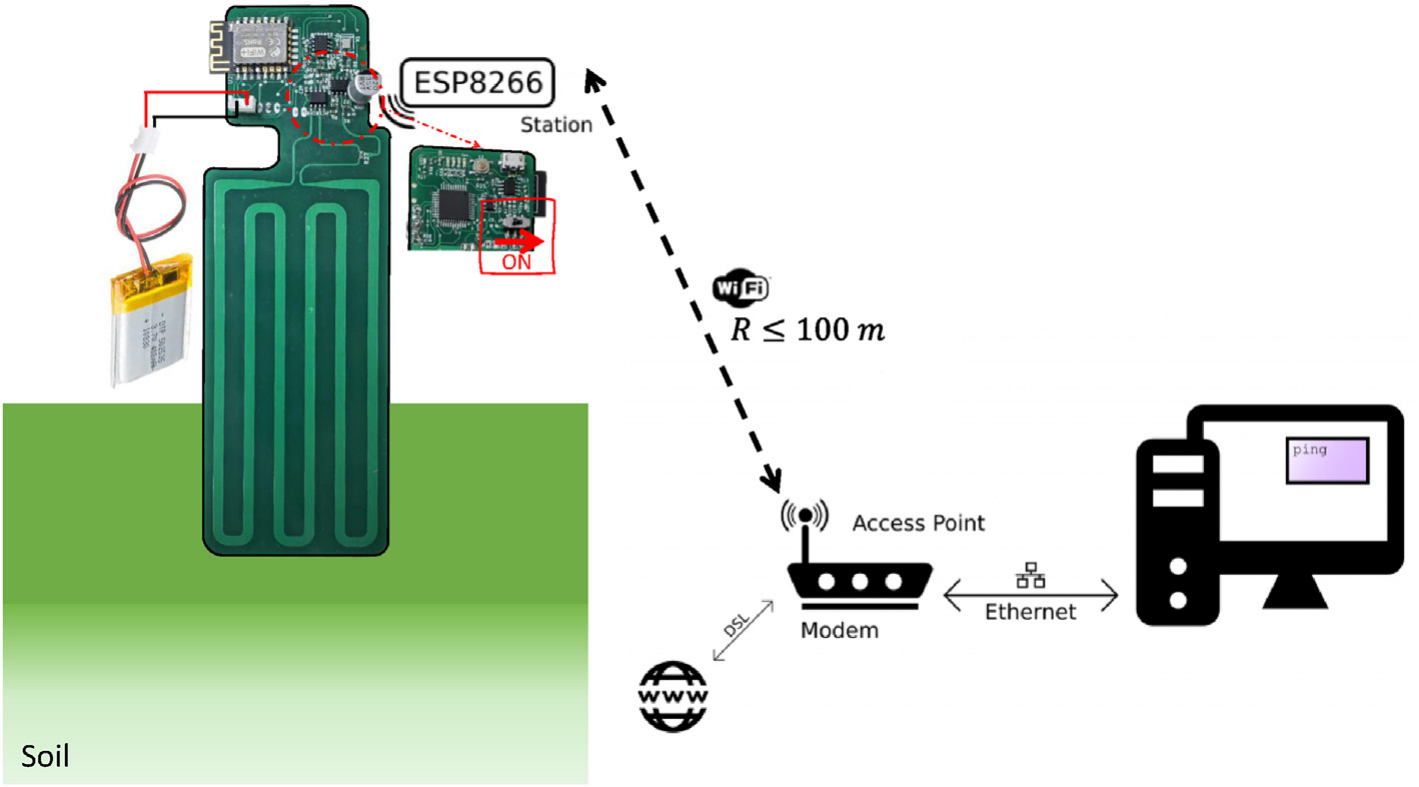
TDT sensor system operation.

The TDT sensor system is designed to have a stand alone operation, it is recommended to install the sensor in a granular soil with low unconfined compressive strength and at no more than 100 meters from the WiFi gateway to guarantee wireless connection with the as it is shown in Fig. 8. Once firmware is loaded and the lion battery is charged and connected, the TDT sensor is ready to operate and report measure data. To check a correct WiFi connection in your computer, open up a terminal: On Windows, search for ”Command Prompt”, on Mac or Linux, search for ”Terminal”. Next, type ping, and then the IP address you received in the Serial monitor after loading the ESP8266 firmware. On the TDT sensor, when WiFI connection is established, the LED blinks twice.

When the TDT sensor system is placed and the slide switch is positioned in ON as indicated in Fig. 8. The TDT sensor will start measuring periodically every 3 min and will report to Ubidots from where all the data is visualized and extracted for post processing analysis. Post-processing is done through the Matlab script PostProcessing_TDT.m included in the Post_Processing folder in the repository. Postprocessing_TDT.m gives the indications for the sensor calibration and computation of the time delay and the dielectric permittivity of SUT.

## 7 Validation and characterization

For validation of the fabricated TDT sensor, in-lab test were carried out. We employed regular garden soil which were first dried in a oven set to 100°C during 8 h. Soil dielectric permittivity greatly varies with the water content, so characterization of the soil permittivity can be directly related to the soil moisture percentage, which was initially proposed by Topp et.al. in [9] and has been widely employed in several works. Some recent research in geophysical and agricultural science show its application [10], [11], [12]. Therefore, our test consists in the measurement of the dielectric permittivity of the soil under test (SUT), with different water content percentages, through our TDT sensor system directly inserted in the soil under analysis, compared with the dielectric permittivity estimation formula given by Topp and the permittivity obtained (Fig. 12) from a calibrated open coaxial probe from a dielectric assessment kit (DAK 3.5 by Speag) [13]. To deal with the sensitivity issues, 5 samples on different parts of the soil were collected and averaged, for each reported value. The volumetric water content Θ of mineral soils can be determined by using Topp formula [9] in (14) which is empirically validated at frequencies between 1 MHz and 1 GHz.

(14) $$\varepsilon \prime_{\mathrm{soil}}=3.03+9.3\;\Theta +146\;\Theta^{2}-76.7\;\Theta^{3}$$

**Fig. 9:**
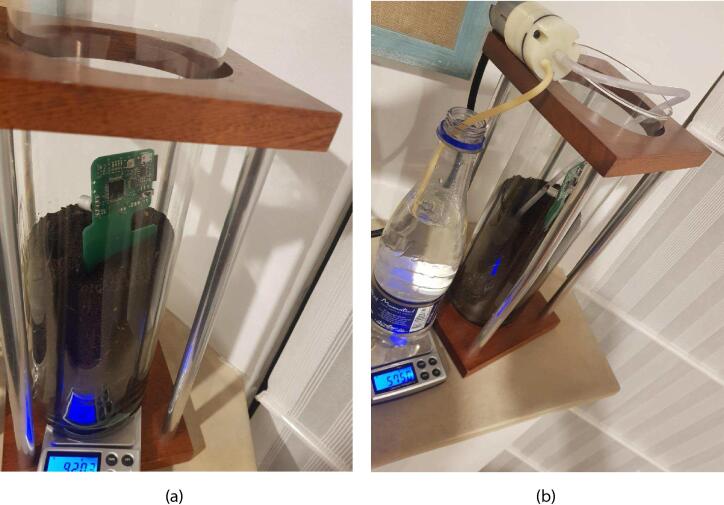
Measurement set-up for TDT sensor system validation.

After the dryer procedure, a sample of the soil was deposited in a cylindrical glass container where the head sensor of the TDT system was introduced. The weight of the SUT was 500 g, obtained through a typical electronic kitchen scale (having at least one decimal place of a gram) as it is shown in Fig. 9a. For increasing the water content inside the SUT a small motor pump was used, as shown in Fig. 9b. The increase was controlled and measured through the electronic scale, and taking as a reference, the initial weight of the SUT, the soil moisture percentage was computed by measuring the weight increase. The motor pump was turned on every 15 min to get the required weight for each moisture target. To guarantee the homogeneity of the SUT, after adding the water, the soil was taken out, mixed and replaced in the glass container before performing each measurement.

**Fig. 10:**
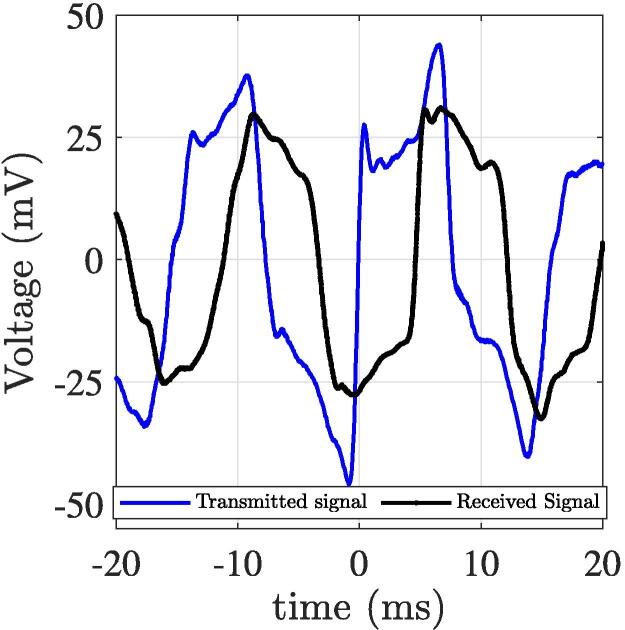
Transmitted and received TDT signal.

**Fig. 11:**
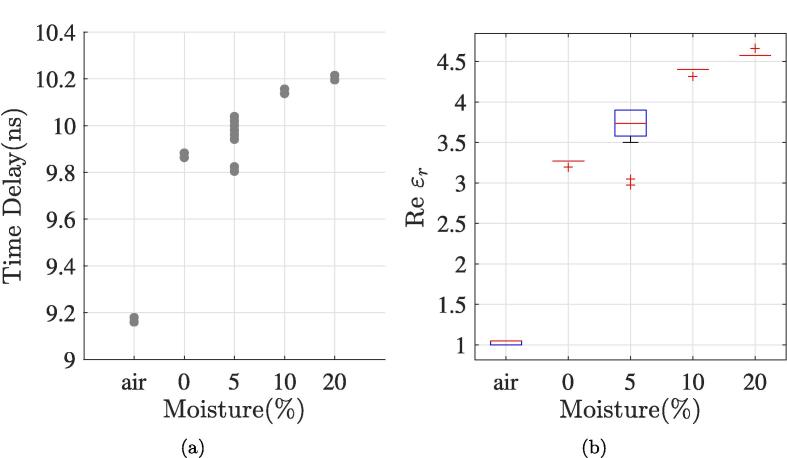
Time delay and box plot of the measured permittivity of the SUT with different water content.

After applying the calibration process, post-processing results in Fig. 11, Fig. 11b show the obtained time delay and dielectric permittivity of the SUT. Table 3 compares the mean measured permittivity obtained with DAK system, the Topp formula and our TDT sensor system. Fig. 10 shows the general characteristics of the transmitted and received TDT signal, with a pulse width of 10 ms and a rising time of 3.8 ms.

**Table 3:** Mean measured permittivity and soil moisture percentage obtained from the DAK and the TDT sensor.

	$\overset{¯}{\varepsilon^{\prime }}$			Mean Absolute Error	
SUT	DAK	**TDT**	Topp	$\left|{\mathrm{MAE}}_{\mathrm{TDT}}\right|$	$\left|{\mathrm{MAE}}_{\mathrm{Topp}}\right|$
Air	1.02	1.04	-	1.02	-
Dry soil	2.51	3.21	3.03	0.75	0.52
Moisture Θ=5%	2.82	3.78	3.85	1.28	1.03
Moisture Θ=10%	3.12	4.38	5.34	1.26	2.22
Moisture Θ=20%	4.05	4.57	10.11	0.52	6.06

**Fig. 12:**
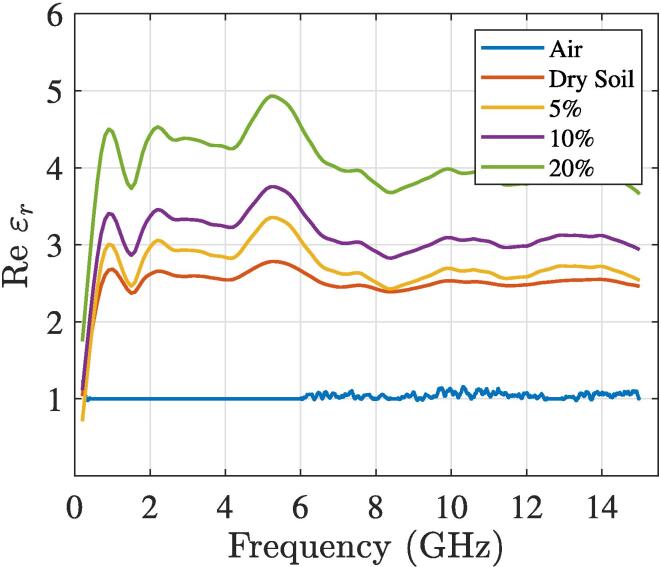
Dielectric soil permittivity measures with different soil moisture percentages, 200MHz-15GHz frequency band with the calibrated DAK.

### 7.1 Discussion

Results obtained from the validation stage shows that our TDT sensor system is a viable low-cost, stand-alone option to measure on-site the dielectric permittivity of granular soils. The results shows that the computed Mean Absolute Error (MAE) when compared with a calibrated dielectric assessment kit (DAK) is less than 1.2 with soils with water content of less than 20%, which also means that our TDT sensor system allows to obtain on-site measurements in relative dry soils with acceptable accuracy. This also validates our hypothesis of obtaining a low-cost, portable, compact, and stand-alone system for dielectric property assessment in soil materials for multiple applications.

### 7.2 Limitations

Our system has a limitation when measuring soils with water contents greater than 20%, that is, soil materials with dielectric permittivities greater than 5, mainly because the selected oscillator frequency is 50 MHz, allowing for maximum time delays of 10 ns. Therefore, future work will focus on improving the performance of the sensor when measuring soil materials with higher permittivity. In this regard, one main fact can be highlighted: Despite the fact that long microstrip transmission lines improve the overall resolution of the sensor, the losses at high frequencies are an issue, especially when measuring dielectric media with high permittivities.

## CRediT authorship contribution statement

**Manuel Pérez:** Conceptualization, Funding acquisition, Project administration, Methodology, Software, Writing - original draft, Visualization, Validation. **Diego Mendez:** Project administration, Data curation, Investigation, Writing - review & editing. **Diego Avellaneda:** Software. **Arturo Fajardo:** Data curation, Writing - review & editing. **Carlos I. Páez-Rueda:** Data curation, Writing - review & editing.

## Declaration of Competing Interest

The authors declare that they have no known competing financial interests or personal relationships that could have appeared to influence the work reported in this paper.

## Footnotes

1 Pocket VNA website:https://pocketvna.com

2 https://data.mendeley.com/datasets/mt24hdxsr2/1
